# Tryptophan Metabolic Pathways Are Altered in Obesity and Are Associated With Systemic Inflammation

**DOI:** 10.3389/fimmu.2020.00557

**Published:** 2020-04-15

**Authors:** Sofia Cussotto, Inês Delgado, Andrea Anesi, Sandra Dexpert, Agnès Aubert, Cédric Beau, Damien Forestier, Patrick Ledaguenel, Eric Magne, Fulvio Mattivi, Lucile Capuron

**Affiliations:** ^1^University of Bordeaux, INRAE, Bordeaux INP, NutriNeuro, UMR 1286, Bordeaux, France; ^2^Department of Food Quality and Nutrition, Research and Innovation Centre, Fondazione Edmund Mach (FEM), San Michele all'Adige, Italy; ^3^Service de Chirurgie Digestive et Parietale, Clinique Tivoli, Bordeaux, and Clinique Jean Villar, Bruges, France; ^4^Department of Cellular, Computational and Integrative Biology (CIBIO), University of Trento, Trento, Italy

**Keywords:** obesity, inflammation, tryptophan, kynurenine, indoles

## Abstract

**Background:** Obesity is a condition with a complex pathophysiology characterized by both chronic low-grade inflammation and changes in the gut microbial ecosystem. These alterations can affect the metabolism of tryptophan (TRP), an essential amino acid and precursor of serotonin (5-HT), kynurenine (KYN), and indoles. This study aimed to investigate alterations in KYN and microbiota-mediated indole routes of TRP metabolism in obese subjects relatively to non-obese controls and to determine their relationship with systemic inflammation.

**Methods:** Eighty-five obese adults (avg. BMI = 40.48) and 42 non-obese control individuals (avg. BMI = 24.03) were recruited. Plasma levels of TRP catabolites were assessed using Ultra High Performance Liquid Chromatography-ElectroSpray-Ionization-Tandem Mass Spectrometry. High-sensitive C-reactive protein (hsCRP) and high-sensitive interleukin 6 (hsIL-6) were measured in the serum as markers of systemic inflammation using enzyme-linked immunosorbent assay.

**Results:** Both KYN and microbiota-mediated indole routes of TRP metabolism were altered in obese subjects, as reflected in higher KYN/TRP ratio and lower 5-HT and indoles levels, relatively to non-obese controls. HsIL-6 and hsCRP were increased in obesity and were overall associated with TRP metabolic pathways alterations.

**Conclusion:** These results indicate for the first time that KYN and indole TRP metabolic pathways are concomitantly altered in obese subjects and highlight their respective associations with obesity-related systemic inflammation.

## Introduction

Obesity is a metabolic disorder characterized by a chronic low-grade inflammatory state, as reflected in increased levels of circulating inflammatory markers, including pro-inflammatory cytokines and the acute phase protein C-reactive protein (CRP) (1–4). Systemic inflammation in obesity originates primarily from the adipose tissue, in which adipocytes and infiltrated immune cells accumulate and secrete inflammatory factors (2–5). Additionally, changes in the gut microbiota composition and permeability that have been highly documented in obesity (6–9), were also found to play a role in obesity-related inflammation (10, 11).

A growing body of evidence indicates that inflammation is associated with alterations in the metabolism of tryptophan (TRP) (12, 13), an essential amino acid and biochemical precursor of serotonin (5-HT), kynurenine (KYN) and indoles (14). One mechanism likely to be involved in this effect relies on the induction of the enzyme indoleamine-2,3-dioxygenase (IDO) by pro-inflammatory cytokines (13, 15). IDO is responsible for the catabolism of TRP along the KYN pathway, likely resulting in reduced 5-HT synthesis. Consistent with this notion, activation of the KYN pathway has been documented in several studies conducted in obese subjects (16–19). Alterations in TRP metabolism in obese subjects may also arise from the gut microbiota, which has been shown to be disrupted both at compositional and functional levels in obesity (20–22). Accordingly, a greater ratio of Firmicutes/Bacteroidetes, along with disrupted intestinal permeability and increased endotoxemia have been repeatedly documented in obese subjects (6–9). Diet and nutritional habits represent the main contributors to obesity-related alterations in the gut microbiota given their major role in shaping intestinal bacteria environment. In the gut, TRP is metabolized by specific intestinal bacteria (23) into indole, indole-3-acetic acid (IAA), indole-3-acrylic acid (IA), indole-3-carboxalaldehyde (ICAld), indole-3-lactic acid (ILA), indole-3-propionic acid (IPA), indole-3-ethanol (IE), indole-3-acetonitrile (IACN), and indole-3-carboxylic acid (ICA) (24). In adults, the most abundant catabolite is indole, followed by IAA and IPA (25–27). Despite strong evidence of gut microbiota changes in obesity, little is known on the impact of these alterations on gut-derived TRP metabolism along the indole pathway.

Altogether, these data suggest that obesity may contribute to concomitant alterations in TRP metabolism, through parallel pathways, involving both inflammation and the microbiota. Moreover, the relationship between these two pathways remains to be determined, in particular the possibility that inflammatory processes relate to indoles production. Supporting this scenario, gut-derived inflammatory processes, including endotoxemia (9), in obesity could disrupt gut homeostasis and function leading thus to substantial alterations in the metabolism of indoles. In addition, indoles represent potent modulators of immune function. They can act as ligands of the aryl hydrocarbon receptor (AHR), a transcription factor widely expressed by cells in the immune system and whose activation can alter innate and adaptive immune responses (28, 29). For instance, ICAld produced by *Lactobacillus* spp. was found to attenuate intestinal inflammation by regulating IL-22 mucosal homeostasis via an AHR-dependant mechanism (30).

The aim of the present study was to investigate the KYN and indole pathways of TRP metabolism in obese subjects relatively to non-obese controls and to determine their relationship with systemic inflammation.

## Methods

### Study Participants

#### Obese Subjects

Eighty-five adult obese subjects with severe or morbid obesity, awaiting a gastric surgery, were recruited from the services of digestive and parietal surgery of two private clinics (Tivoli and Jean-Villar) in Bordeaux, France. Participants met criteria for obesity surgery, i.e., BMI ≥ 40 kg/m^2^ or ≥ 35 kg/m^2^ with at least one comorbidity [e.g., hypertension [HT], type-2 diabetes [T2D], obstructive sleep apnea [OSA], dysthyroidism].

#### Non-obese Controls

Forty-two non-obese volunteers (BMI <30 kg/m^2^) with no acute or chronic immune/inflammatory condition were included as control participants. A level of high-sensitive (hs) CRP above 5 mg/L, indicative of low-grade inflammation (31), was considered as an exclusion criterion in this group of participants. Control subjects were recruited by phone interview conducted by the contract research organization CEN Nutriment (Dijon, France).

In both groups, exclusion criteria were: age > 65 years old; acute or chronic inflammatory conditions (other than obesity or obesity-related comorbidities); diagnosis of severe or uncontrolled medical illness; and current treatment with anti-inflammatory agents. All participants provided written informed consent after reading a complete description of the study. The study was approved by the Institutional Committee of Protection of Persons (CPP; registration numbers 2010/36 and 2016/40 for obese and non-obese subjects, respectively).

### Measurements

#### Socio-Demographic and Clinical Characteristics

Socio-demographic and clinical characteristics, including anthropometric data, medical history and current treatment, were collected by trained professionals for all participants at inclusion. BMI was calculated as weight (kg)/height (m)^2^.

#### Biological Measurements

The same day as clinical assessments, fasting blood samples were collected in plain or EDTA-containing tubes for serum and plasma, respectively. After 30–45 min at room temperature, samples were centrifuged (4,000 rpm, 20 min at 4°C for plasma and 3,200 rpm, 10 min at 4°C for serum) and stored at −80°C until further analysis.

#### Inflammatory Markers

Serum concentrations of hsCRP and high-sensitive interleukin 6 (hsIL-6) were determined by enzyme-linked immunosorbent assay (ELISA) according to the manufacturer's specifications (hsCRP: CYT298, Millipore, Billerica, Massachusetts; hsIL-6: R&D Systems, Minneapolis, Minnesota). Assays sensitivity and intra-/inter-assay variability were, respectively, 0.20 ng/mL, ± 4.6% and ± 6.0% for hsCRP and 0.031 pg/mL, ± 4.1% and ± 3.9% for hsIL-6.

#### Tryptophan Metabolites

Plasma concentrations of free TRP, 5-HT, 5-hydroxyindole-3-acetic acid (5-HIAA), KYN, IAA, ICAld, ILA, IPA, and indoxyl sulfate (IS) were determined by Ultra High Performance Liquid Chromatography-ElectroSpay-Ionization-Tandem Mass Spectrometry (UHPLC-ESI-MS/MS), as described in detail elsewhere (32). This technique was developed and validated for the targeted quantification of TRP and tyrosine derived metabolites in human plasma and urine. The ratio KYN/TRP was calculated as an index of TRP breakdown along the KYN pathway indicative of IDO activation.

### Data Analysis

Raw values for serum inflammatory markers as well as for plasma levels of TRP, 5-HT, 5-HIAA, IAA, ICAld, ILA, IPA, and IS were log-transformed due to non-normality, as assessed by the Shapiro-Wilk test. Extreme values (>3SD above the mean) were observed for IL-6 (*N* = 1 non-obese participant), 5-HIAA (*N* = 1 obese participant) and IAA (*N* = 2 obese participants). Accordingly, these values were considered outliers and were individually excluded from data analyses performed on biological markers. Of note, values for hsIL-6 were missing in two non-obese controls. Socio-demographic and clinical characteristics of the two experimental groups were compared using Student's *t*-tests for continuous variables or Chi-square tests for categorical variables. Levels of hsCRP, hsIL-6, TRP, and markers of its metabolic pathways were compared between the two groups using analyses of covariance (ANCOVAs) controlling for age, gender, and comorbidities. The relationship between inflammatory markers, BMI, and TRP metabolism (KYN/TRP and indoles) was estimated in the whole population under study and separately in the obese group using multiple regression analyses, adjusted for age, gender, and comorbidities. Statistical analyses were performed with SPSS Statistics version 25. All probabilities were two-sided with the degree of significance set at *p* < 0.05.

## Results

### Socio-Demographic and Clinical Characteristics of Study Participants

Socio-demographic and clinical characteristics of study participants are presented in Table 1. There were no significant differences between obese subjects and non-obese controls in terms of age (*t* = 0.410, *p* = 0.683) and gender (Chi^2^ = 0.144, *p* = 0.705). As expected, BMI and weight were significantly higher in the obese group compared to the non-obese one (BMI: *t* = 22.48, *p* < 0.0001; weight: *t* = 16.62, *p* < 0.0001). Similarly, the prevalence of HT was higher in obese subjects (Chi^2^ = 8.451, *p* =0.004), as well as T2D and OSA that were only present in the obese population.

**Table 1:** Characteristics of study participants.

	**Non-obese participants**	**Obese participants**	***p***
Sample size, n	42	85	
Age, years (SD)	37.74 (7.48)	38.52 (111.14)	0.683
Women, n (%)	35 (83.33)	73 (85.88)	0.705
BMI, kg/m^2^ (SD)	24.03 (3.52)	40.48 (4.05)	<0.0001
Weight, kg (SD)	68.18 (11.55)	111.20 (14.68)	<0.0001
**Comorbidities**			
HTA, n (%)	1 (2.38)	19 (22.35)	0.004
T2D, n (%)	0 (0)	10 (11.77)	0.021
Dyst, n (%)	1 (2.38)	9 (10.59)	0.106
OSA, n (%)	0 (0)	27 (45.0)	<0.001

*Continuous variables are presented as mean and standard deviation and compared using Student's t-test, whereas categorical variables are presented as n (%) and compared by chi-square test. BMI, body mass index; HTA, hypertension; T2D, type 2 diabetes; Dyst, dysthyroidism; OSA, obstructive sleep apnea*.

### TRP Metabolic Pathways Are Altered in Obese Subjects Compared to Healthy Participants

**Figure 1:**
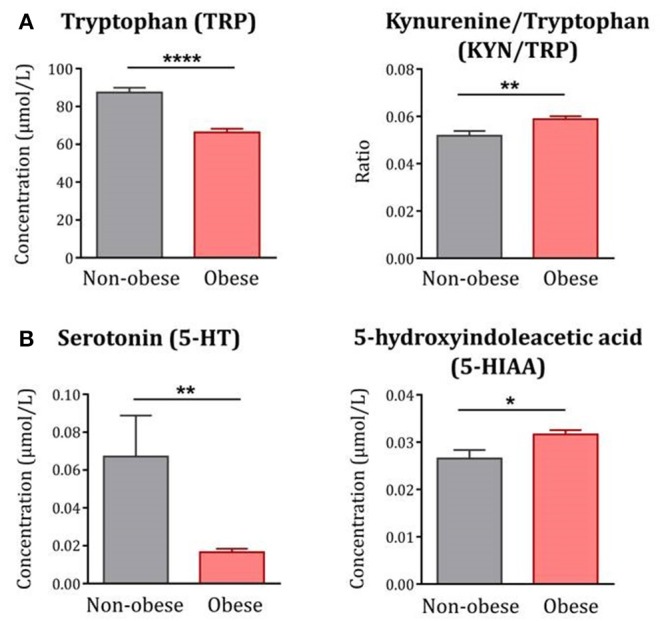
TRP metabolism along the KYN and 5-HT pathways in obese subjects compared to non-obese controls. **(A)** Plasma levels of TRP were decreased whereas the KYN/TRP ratio was increased in obese subjects (*n* = 85) compared to non-obese controls (*n* = 42). **(B)** Similarly, plasma levels of 5-HT were decreased whereas 5-HIAA was increased in obese subjects (*n* = 84–85). Statistical analyses were performed on log-transformed data using ANCOVA controlling for age, gender and comorbidities. Data are expressed as mean + SEM. ^****^p < 0.0001, ^**^p < 0.01, ^*^p < 0.05.

Compared to non-obese controls, obese subjects exhibited decreased circulating levels of TRP [F_(1, 122)_ = 37.79*, p* < *0.0001*] together with increased KYN/TRP ratio [F_(1, 122)_ = 9.77*, p* < *0.01*], indicative of IDO activation and TRP breakdown along the KYN pathway (Figure 1A). In line with the impact of this pathway on 5-HT synthesis, plasma levels of 5-HT were significantly decreased in obese subjects [F_(1, 122)_ = 10.83*, p* < *0.01*] whereas levels of 5-HIAA [F_(1, 121)_ = 5.57*, p* < *0.05*] were increased (Figure 1B).

**Figure 2:**
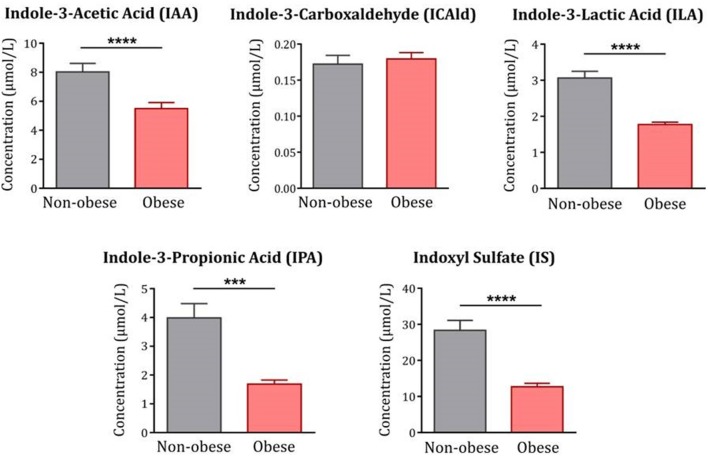
Indole metabolites in obese subjects compared to non-obese controls. With the exception of ICAld, all indoles were significantly decreased in obese subjects (*n* = 83–85) compared to non-obese controls (*n* = 42). Statistical analyses were performed on log-transformed data using ANCOVA controlling for age, gender and comorbidities. Data are expressed as mean + SEM. ^****^p < 0.0001; ^***^p < 0.001.

Moreover, levels of IAA, ILA and IPA were all significantly decreased in obese subjects compared to non-obese controls [IAA: F_(1, 120)_ = 19.53*, p* < *0.0001;* ILA: F_(1, 122)_ = 64.34*, p* < *0.0001;* IPA: F_(1, 122)_ =13.89*, p* < *0.001*] (Figure 2). In contrast, levels of ICAld were not altered [F_(1, 122)_ = 0.29, *p* = *0.59*]. IS, an indole-derived metabolite produced in the liver from free indole, was also significantly decreased in the obese group [(F_(1, 122)_ = 21.83*, p* < *0.0001*].

Consistent with these results, BMI was positively associated with KYN/TRP ratio and 5-HIAA, but negatively correlated with levels of IAA, ILA, IPA, IS, TRP, and 5-HT in the whole population under study (data not shown).

### Systemic Inflammation Correlates With Alterations in TRP Metabolic Pathways

**Figure 3:**
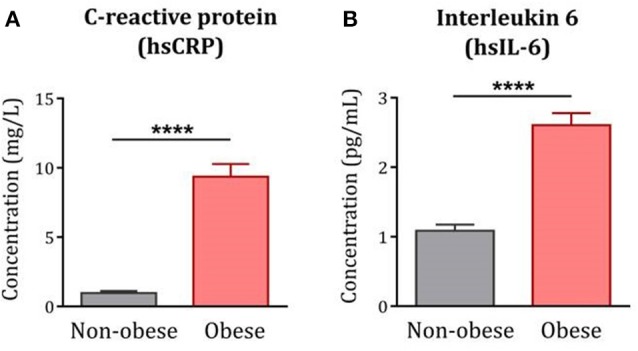
Systemic inflammation in obese subjects compared to non-obese controls. Serum levels of hsCRP and hsIL-6 were significantly increased in the obese population (*n* = 85) compared to non-obese controls (*n* = 39–42). Statistical analyses were performed on log-transformed data using ANCOVA controlling for age, gender and comorbidities. Data are expressed as mean + SEM. ^****^p < 0.0001. *hsCRP*, high-sensitive C-reactive protein; *hsIL-6*, high-sensitive interleukin-6.

As expected, serum levels of hsCRP [F_(1, 122)_ = 102.50*, p* < *0.0001*] and hsIL-6 [F_(1, 119)_ = 54.71*, p* < *0.0001*] were increased in obese subjects compared to non-obese controls (Figure 3). In line with this, hsCRP and hsIL-6 were positively associated with BMI in the whole population under study and were correlated to each other (data not shown). Increased levels of hsCRP and hsIL-6 were associated with reduced levels of TRP together with increased KYN/TRP ratios in the whole population, consistent with the inflammatory characteristic of this pathway. No significant correlations were found between inflammation and markers of 5-HT pathway (Table 2).

**Table 2:** Association between hsCRP, hsIL-6, and markers of tryptophan metabolism in the whole population.

	**TRP**	**KYN/TRP**	**5-HT**	**5-HIAA**	**IAA**	**ICAld**	**ILA**	**IPA**	**IS**
hsCRP	−0.397^****^	0.190^*^	−0.088	0.133	−0.332^***^	−0.078	−0.501^****^	−0.268^**^	−0.339^***^
hsIL-6	−0.380^****^	0.300^**^	−0.158	0.068	−0.290^**^	−0.108	−0.397^****^	−0.244^**^	−0.304^***^

Multiple regression analyses controlling for gender, age and comorbidities (β coefficient). IAA, indole-3-acetic acid; ICAld, indole-3-carboxaldehyde; ILA, indole-3-lactic acid; IPA, indole-3-propionic acid; IS, indoxyl sulfate; KYN/TRP, kynurenine/tryptophan; 5-HT, serotonin; 5-HIAA, 5-hydroxyindole-3-acetic acid.

**** p < 0.0001,

*** p < 0.001,

** p <0.01,

* *p < 0.05*.

Interestingly, both hsCRP and hsIL-6 levels were negatively associated with plasma levels of IAA, ILA, IPA, and IS (Table 2). While no significant correlations were observed between inflammatory markers and ICAld levels in the whole population under study, this metabolite was significantly correlated with hsIL-6 levels in the group of obese subjects (β = −0.261, *p* < 0.05, data not shown).

## Discussion

There is an increasing emphasis on the interplay between obesity, inflammation, and TRP metabolism. While most of the research in inflammatory conditions has been focused on the KYN route of TRP metabolism, notably through activation of the enzyme IDO, here we assessed whether obesity-related inflammation may also impact the microbial route of TRP metabolism. To our knowledge, this is the first study showing concomitant alterations in the KYN and indole pathways of TRP metabolism in obesity.

In line with previous reports (17, 19), obese subjects exhibited higher serum levels of inflammatory markers together with reduced plasma levels of TRP and increased KYN/TRP ratio, indicative of inflammation-driven IDO activation. Interestingly, and consistent with the inflammatory component of the KYN pathway, the ratio of KYN/TRP was significantly associated with levels of systemic inflammation in the whole population under study. Alterations were also found in plasmatic markers of the 5-HT pathway, with obese subjects exhibiting reduced 5-HT and increased 5-HIAA levels. These findings are consistent with the hypothesis of an IDO-driven shift of TRP degradation toward the KYN pathway at the detriment of 5-HT pathway in obesity.

In addition, our results indicate that obesity is associated with significant reductions in microbial-derived indoles, notably IAA, ILA, and IPA. These alterations may rely on changes in microbiota composition and function, as described in obesity. Similarly, IS, an indole-derived metabolite produced in the liver, was also decreased in obese subjects, probably due to the low availability of its substrate, indole. Supporting this notion, reduced indole levels have been documented in children with class II-III obesity when compared to their healthy counterparts (33). Interestingly, findings from the present study reveal significant relationships between circulating levels of indoles and inflammatory markers. In particular, plasma levels of IPA, IAA, ILA, and IS were negatively correlated with serum levels of hsCRP and hsIL-6 in the whole population under study. Noteworthy, ICAld was the only indole metabolite that was not affected by obesity *per se* but showed an association with inflammatory markers, especially hsIL-6, in the group of obese subjects. The nature of this association needs to be disentangled in future studies. To our knowledge, this report is the first to demonstrate a concomitant alteration of host (KYN) and microbial (indole) TRP metabolic pathways, both relating on systemic inflammation, in obesity. While inflammation is known to induce KYN pathway activation (13, 34), the direction of its relationship with the indole pathway is unclear and remains to be investigated in future studies. Data from the literature is heterogeneous and suggests that this relationship may be bidirectional. Not only indoles have been described as potent regulators of immune/inflammatory processes (30, 35, 36) but also gut inflammation, often described in obesity (6–9), is likely to modify microbiota-driven indoles production.

The present study bears some limitations. First, we did not assess fecal levels of indoles nor perform a microbiota characterization of study participants, which would add an important piece of data to this work. Second, only hsCRP and hsIL-6 were measured as markers of systemic inflammation. Although those markers are probably the most used in the literature to evaluate chronic low-grade inflammation (37), a more comprehensive spectrum of inflammatory factors is needed to assess the influence of obesity-related inflammation on microbial TRP metabolism. Finally, no record on food consumption was available in the study. Since diet is a key source of TRP (38), this information would help clarifying to what extent the differences in TRP levels observed between obese and non-obese subjects are linked to different dietary habits.

In conclusion, the present study demonstrates for the first time concomitant alterations in KYN and indole TRP metabolic pathways in obese subjects and highlights their respective associations with obesity-related systemic inflammation. These findings point to TRP metabolism as an important component of obesity. While the relevance of this mechanism to the pathophysiology and management of obesity and its comorbidities remains to be determined, this opens new avenues for future research.

## Data Availability Statement

The datasets generated for this study are available on request to the corresponding author.

## Ethics Statement

The studies involving human participants were reviewed and approved by CPP Bordeaux. The patients/participants provided their written informed consent to participate in this study.

## Author Contributions

SC, ID, and LC contributed to the conception and/or design of the study and were involved in writing the manuscript. CB, DF, PL, and EM enrolled obese participants in the study and performed the medical examinations. SD performed study inclusions and was involved in patients' follow up. ID and AAu conducted the biological experiments. SC and ID performed data analysis. AAn and FM assisted with the target metabolomics and interpretation of results. All authors critically revised the manuscript and approved the final version.

### Conflict of Interest

The authors declare that the research was conducted in the absence of any commercial or financial relationships that could be construed as a potential conflict of interest.
